# Evaluation of upper airway ultrasonographic measurements in predicting difficult intubation: A cross-section of the Turkish population

**DOI:** 10.1515/med-2024-1013

**Published:** 2024-08-21

**Authors:** Tugba Nur Sayir, Bilge Tuncer, Ezgi Erkilic

**Affiliations:** Ankara City Hospital, Anesthesiology and Reanimation, Ankara, Turkey

**Keywords:** ultrasonography, neck circumference/thyromental distance, difficult intubation

## Abstract

**Objectives:**

Studies have shown that there are differences in clinical evaluation parameters and difficult intubation rates among different ethnic populations. In our study, we aimed to evaluate the efficacy of upper airway clinical and ultrasonographic measurement methods in Turkish population.

**Methods:**

Our study is a single-center, prospective, observational study conducted with 402 patients. All patients underwent clinical airway measurements which are routinely used in pre-anesthetic evaluation. In addition, ultrasonographic anterior neck soft tissue thickness measurements of each patient were made and recorded.

**Results:**

Among the clinical measurements, we found the neck circumference/thyromental distance (TMD) ratio to be significant with a cut-off value of 5.5 and a sensitivity of 92.9% and a specificity of 88.3%, while among the ultrasonographic anterior neck measurements, we found the skin–epiglottic distance to be the most sensitive measurement. We found that there was a positive relationship between the neck circumference/TMD ratio and skin–epiglottis.

**Conclusions:**

In our study, we found that routine measurement methods used in airway examination alone are not sufficient, and measurements that take into account the body proportions of the patients, such as the neck circumference/TMD ratio and the ultrasonographic evaluations are more useful in predicting difficult intubation.

## 1 Introduction

Airway management is a very important issue in terms of the safety of perioperative anesthesia. Difficult ventilation and difficult intubation are important causes of anesthesia-related perioperative morbidity and mortality. It has been reported that approximately 30% of anesthesia-related deaths are associated with inadequate management of difficult airway [1,2].

Airway assessment constitutes an important part of the pre-anesthesia evaluation to identify patients who may encounter difficult intubation and difficult ventilation [3].

Routine clinical features that are evaluated for the risk prediction of difficult airway include age, gender, body mass index (BMI), weight, height, history of difficult intubation, abnormal airway anatomy, snoring, obstructive sleep apnea (OSA), measurement of facial and jaw features, mouth opening, prognathic ability, head and neck mobility, prominent upper incisors, beard presence, upper lip bite test (ULBT), Mallampati score, thyromental distance (TMD), sternomental distance, inter-incisor distance, neck circumference, hyomental distance, ratio of neck circumference to TMD, and ratio of neck to TMD.

Routine clinical parameters are valuable in predicting a difficult airway; however, studies have shown that they do not have sufficient negative predictive value to exclude a difficult airway. Clinicians may be faced with unexpected difficult laryngoscopy despite clinical airway examination that suggests otherwise [1].

The use of ultrasonography in preoperative airway evaluation has become widespread with the developing technology and increased use of ultrasonography in clinics. It is a real-time, non-invasive, easily accessible, mobile, safe, and painless diagnostic tool that can be used in the evaluation of both upper and lower airway [4]. Measurements from ultrasonography include skin–vocal cord distance, skin–hyoid distance, tongue volume, and skin–epiglottis distance [5].

It has been observed in studies that there are differences in clinical evaluation parameters and rates of difficult intubation between different ethnic populations [6–8]. Anthropometric studies have reported ethnic variations in body structure and morphometry and morphology of craniofacial bones (maxilla and mandible bones) [9]. Anatomical changes related to ethnic differences also significantly affect the risk of OSA [10].

In this study, which we thought could reflect the Turkish population, we aimed to determine and compare the efficacy of clinical and ultrasonographic measurement methods of the upper airway used in preoperative airway assessment in predicting difficult intubation and laryngoscopy. We also aimed to determine the specificity and sensitivity of these measurement methods for difficult intubation and laryngoscopy, and to find threshold values for the Turkish population. Our secondary aim in this study was to determine the rates of difficult intubation and difficult laryngoscopy in patients with OSA in the population.

## 2 Materials and methods

For this single-center, prospective, observational study, 402 patients aged between 18 and 65 years with ASA 1–2 characteristics who would undergo elective surgery under general anesthesia were included after obtaining the approval of the ethics committee. Patients who were pregnant, patients with neck mobility limitation (such as rheumatologic diseases, masses), cervical spine pathologies, masses and lesions in the mouth and airway that would make intubation difficult, cervical and maxillofacial trauma, a history of radiotherapy to the neck, subglottic stenosis, nasotracheal intubation, and double lumen tube use were excluded.

Weight (kg), height (cm), age (year), gender, BMI values, and ASA score were recorded after obtaining the written informed consent of the patients during preoperative evaluation. After the Mallampati score, ULBT and loose teeth were determined, neck circumference, TMD, sternomental distance, and mouth opening were measured. The results of STOP-Bang scoring which consists of eight questions and evaluated with yes/no answers to the questions were noted.

The patients were grouped into OSA risk classes (low-level OSA risk yes answer to 0–2 questions, moderate OSA risk yes answer to 3–4 questions, high-level OSA risk yes answer to 5–8 questions [or yes answer to 2 or more of 4 STOP questions + male participant or yes answer to 2 or more of 4 STOP questions + BMI >35 kg/m² or yes answer to 2 or more of 4 STOP questions + increased neck circumference]).

Afterward, the patients were placed in supine, neck flexion, head extension (sniffing-sniffing) position and prepared for ultrasonographic evaluation. Skin–hyoid bone, skin–epiglottis, skin–epiglottis, skin–vocal cord anterior commissure distance measurements were made and recorded with the 13–6 MHz linear probe of USG (SonoSite S-Nerve).

All patients underwent routine ASA monitoring (ECG, NIBP, SpO_2_) followed by pre-oxygenation before induction of general anesthesia. Anesthesia induction was chosen according to the practice of the clinic and the clinical status of the patients. After the appropriate muscle relaxant was administered and adequate muscle relaxation was achieved, orotracheal intubation was performed with the most appropriate number of tubes for each patient. Intubation was performed by an experienced anesthesiologist. The auxiliary stylet used during intubation, the need for cricoid compression, the number of extra attempts, the number of extra practitioners, the need for mandibular suspension, and the glottis patency without external compression seen during laryngoscopy were noted in accordance with the Cormack Lehane Grade. Advanced airway devices (video laryngoscopy and flexible fiber optic laryngoscopy) used when needed were recorded. Intubation Difficulty Scores (IDS) were calculated, and patients were divided into groups as “Easy” if IDS was 0, “Partially easy” if 0 < IDS > 5, and “Difficult” if IDS > 5.

### 2.1 Statistical analysis

Data analysis was performed using IBM SPSS 25.0 (Armonk, NY: IBM Corp.) and MedCalc 15.8 (MedCalc Software bvba, Ostend, Belgium) statistical package programs. Descriptive statistical methods (frequency, percentage, mean, standard deviation, median, min–max) and Chi-square (*χ* ^2^) test were used to compare qualitative data. The conformity of the data to normal distribution was evaluated by Kolmogorov–Smirnov test, skewness and kurtosis, and graphical methods (histogram, Q–Q Plot, Stem and Leaf, Boxplot). Independent samples *t*-test (*t-*test in independent groups) was used in the evaluation of quantitative data showing normal distribution. Receiver operating characteristic (ROC) curve method was used to determine the discrimination of variables. Pearson correlation test and Spearman’s Rho correlation test were used to evaluate the relationships between variables. Statistical significance level was accepted as *α* = 0.05.


**Ethical approval:** Approval of the ethics committee dated 15.03.2023 and numbered E2-23-3585 at the Ministry of Health Ankara City Hospital.
**Informed consent:** Informed consent has been obtained from each patient.

## 3 Results

Our study included a total of 402 patients, 183 female (45.5%) and 219 (54.5%) male, aged between 18 and 65 years. Demographic characteristics of the participants and routine screening tests are shown in Tables 1 and 2.

**Table 1:** Demographic characteristics of participants

		*n* Mean ± SD	% Median (min–max)
Gender*	Female	183	45.5
	Male	219	54.5
Age (year)**		44.1 ± 14.2	45.0 (18.0–65.0)
Weight (kg)**		79.4 ± 10.9	78.0 (53.0–117.0)
Height (cm)**		168.8 ± 8.1	169.0 (154.0–190.0)
BMI (kg/m^2^)**		27.9 ± 3.5	27.7 (20.0–42.2)
	<30*	*289*	*71.9*
	≥30*	*113*	*28.1*
CPAP at home*	No	394	98.0
	Yes	8	2.0
Stop-Bang score*	No OSA	337	83 > 8
	Moderate OSA risk	32	8.0
	High OSA risk	33	8.2

*n/%; **mean ± standard deviation/median (min–max).

**Table 2:** Comparison of study participants according to routine tests

		*n* Mean **±** SD	% Median (min–max)
Mallampati score*	1	96	23.9
	2	294	73.1
	3	11	2.7
	4	1	0.2
Mallampati intubation*	*Easy*	*390*	*97.0*
	*Difficult*	*12*	*3.0*
ULBT*	Grade1 (easy)	288	71.6
	Grade 2 (partially difficult)	83	20.6
	Grade 3 (difficult)	31	7.7
TMD (cm)**		7.2 ± 0.5	7.3 (5.6–8.3)
Sternomental distance (cm)**		14.1 ± 0.8	14.0 (11.0–16.5)
Neck circumference (cm)**		38.0 ± 1.8	37.8 (34.0–46.0)
Height/TMD ratio**		23.4 ± 1.7	23.2 (19.2–29.2)
Neck circumference/TMD ratio**		5.3 ± 0.5	5.1 (4.3–7.6)
Skin–hyoid bone (cm)**		1.0 ± 0.1	1.0 (0.5–1.4)
Skin–epiglottis (cm)**		2.0 ± 0.3	2.0 (1.0–2.9)
Skin–anterior vocal cord (cm)**		1.0 ± 0.1	1.0 (0.7–1.9)

*n/%; **mean ± standard deviation/median (min–max).

Table 3 shows the comparison of demographic characteristics according to the level of intubation difficulty. In the comparisons between the groups, it was found that there was no statistically significant difference between the groups in terms of gender and age (*p* > 0.05), while there was a statistically significant difference between the groups in terms of weight, height, and BMI values (*p* < 0.05). It was found that the patient group with a Difficult IDS result had lower height values and higher weight and BMI values (BMI ≥ 30).

**Table 3:** Comparison of demographic characteristics according to IDS

IDS				
		Easy (*n* = 360)	Difficult (*n* = 42)	*p*
Gender	Female	160 (44.4%)	23 (54.8%)	0.268^a^
	Male	200 (55.6%)	19 (45.2%)	
Age (year)		44.1 ± 14.3	44.3 ± 12.8	0.928^b^
Weight (kg)		78.4 ± 10.3	88.1 ± 12.3	**<0**.**001** ^b^
Height (cm)		169.1 ± 8.0	165.9 ± 8.2	**0**.**014** ^b^
BMI (kg/m^2^)		27.4 ± 3.1	32.1 ± 3.8	**<0**.**001** ^b^
	*<30*	*280 (77*.*8%)*	*9 (21*.*4%)*	***<0***. ***001*** ^a^
	*≥30*	*80 (22*.*2%)*	*33 (78*.*6%)*	

^a^Chi-square test (*n* (%)); ^b^Independent samples *t*-test (mean ± SD).

Bold means significant *p* values. Italıc words means subgroup.

Intergroup comparisons showed that there was a statistically significant difference between the groups in terms of Mallampati score values (*p* < 0.05), and the patient group with Difficult Intubation Score Difficult had higher rates of Mallampati score 3–4.

In intergroup comparisons, it was found that there was a statistically significant difference between the groups in terms of ULBT values (*p* < 0.05), and the patient group with Difficult Intubation Score Difficult had higher rates of ULBT Grade 2–3. In intergroup comparisons, it was found that there was a statistically significant difference between the groups in terms of Stop-Bang Score values (*p* < 0.05), and the patient group with Difficult Intubation Score was found to have higher rates of high-level OSA risk.

In intergroup comparisons, a statistically significant difference (*p* < 0.05) was found between the groups in terms of all variables. It was found that thyromental and sternomental distance values were lower, while neck circumference, height/TMD ratio, neck circumference/TMD ratio, skin–hyoid bone, skin–epiglottis, and skin–anterior vocal cord values were higher in the patient group with Difficult IDS (Table 4).

**Table 4:** Comparison of distances and rates according to IDS

IDS			
	Easy (*n* = 360)	Difficult (*n* = 42)	p*
TMD (cm)	7.3 ± 0.4	6.5 ± 0.5	**<0**.**001**
Sternomental distance (cm)	14.2 ± 0.7	13.2 ± 1.1	**<0**.**001**
Neck circumference (cm)	37.7 ± 1.4	40.8 ± 2.0	**<0**.**001**
Height/TMD ratio	23.1 ± 1.5	25.6 ± 1.8	**<0**.**001**
Neck circumference/TMD ratio	5.2 ± 0.4	6.3 ± 0.6	**<0**.**001**
Skin–hyoid bone (cm)	0.96 ± 0.14	1.08 ± 0.16	**<0**.**001**
Skin–epiglottis (cm)	1.99 ± 0.21	2.49 ± 0.23	**<0**.**001**
Skin–anterior vocal cord (cm)	0.99 ± 0.13	1.11 ± 0.17	**<0**.**001**

*Independent samples *t*-test (mean ± SD).

Bold means significant *p* values.

Around 396 (98.5%) patients were intubated with a Macintosh plate and 6 (1.5%) with a video laryngoscope. Among the patients with difficult airway, 37 (88.1%) patients were intubated with a Macintosh plate and 5 (11.9%) patients were intubated with a video laryngoscope. Around 326 (81%) patients were intubated on the first intubation attempt and 72 (17%) patients were intubated on the second attempt.

There was no statistically significant difference (*p* > 0.05) between the groups in terms of neck circumference/TMD ratio, skin–hyoid bone, skin–epiglottis, and skin–vocal cord anterior, while there was a statistically significant difference (*p* < 0.05) between the groups in terms of TMD, sternomental distance, neck circumference, and height/TMD ratio. In all cases where there was a difference, the values of males were found to be higher.

In the intergroup comparisons, it was found that there was a statistically significant difference (*p* < 0.05) between the groups in terms of all variables. It was found that the patient group with BMI ≥30 had lower thyromental and sternomental distance values and higher neck circumference, height/TMD ratio, neck circumference/TMD ratio, skin–hyoid bone, skin–epiglottis, and skin–vocal cord anterior values.

There was a statistically significant difference (*p* < 0.05) between the groups in terms of all variables. It was found that the thyromental and sternomental distance values were lower, while neck circumference, height/TMD ratio, neck circumference/TMD ratio, skin–hyoid bone, skin–epiglottis, and skin–vocal cord anterior values were higher in the patient group with difficult Cormack Lehane Grade.

As a result of the evaluations made with ROC analysis (Table 5) the variables were found to be different in the comparisons made according to the IDS:

– For weight values, >78 was found to be the cut-off point (AUC = 0.734, *p* < 0.001, 95% CI: 0.688–0.776).
– For height, ≤168 was found to be the cut-off point (AUC = 0.623, *p* = 0.006, 95% CI: 0.574–0.671).
– In BMI values, >30.7 was found to be the cut-off point (AUC = 0.83, *p* < 0.001, 95% CI: 0.790–0.865).
– In TMD values, ≤6.8 was found to be the cut-off point (AUC = 0.881, *p* < 0.001, 95% CI: 0.845–0.911).
– For sternomental distance values, ≤13.2 was found to be the cut-off point (AUC = 0.797, *p* < 0.001, 95% CI: 0.754–0.835).
– For neck circumference values, >38.7 was found to be the cut-off point (AUC = 0.909, *p* < 0.001, 95% CI: 0.876–0.935).
– In neck circumference/TMD ratio values, >5.5 was found to be the cut-off point (AUC = 0.94, *p* < 0.001, 95% CI: 0.912–0.961).
– In the height/TMD ratio values, >24.25 was found to be the cut-off point (AUC = 0.851, *p* < 0.001, 95% CI: 0.812–0.884).
– In skin–hyoid bone values, >0.99 was found to be the cut-off point (AUC = 0.708, *p* < 0.001, 95% CI: 0.661–0.752).
– In skin–epiglottis values, >2.23 was found to be the cut-off point (AUC = 0.923, *p* < 0.001, 95% CI: 0.892–0.947).

**Table 5:** ROC analysis

	AUC	95% CI	Cut-off	Sensitivity	Specificity	Youden index	+PV	−PV	p*
Weight (kg)	0.734	0.688–0.776	**>78**	85.7	55.0	0.407	18.2	97.1	**<0**.**001**
Height (cm)	0.623	0.574–0.671	**≤168**	66.7	54.4	0.211	14.6	93.3	**0**.**006**
BMI (kg/m^2^)	0.830	0.790–0.865	**>30**.**7**	73.8	85.6	0.594	37.3	96.6	**<0**.**001**
TMD (cm)	0.881	0.845–0.911	**≤6**.**8**	76.2	89.4	0.656	45.7	97.0	**<0**.**001**
Sternomental distance (cm)	0.797	0.754–0.835	**≤13**.**2**	61.9	**92**.**8**	0.547	50.0	95.4	**<0**.**001**
Neck circumference (cm)	0.909	0.876–0.935	**>38**.**7**	90.5	83.6	0.741	39.2	98.7	**<0**.**001**
Height/TMD ratio	**0**.**940**	0.912–0.961	**>5**.**5**	**92**.**9**	88.3	0.812	48.1	99.1	**<0**.**001**
Neck circumference/TMD ratio	0.851	0.812–0.884	**>24**.**25**	81.0	79.7	0.607	31.8	97.3	**<0**.**001**
Skin–hyoid bone (cm)	0.708	0,661–0.752	**>0**.**99**	66.7	70.6	0,372	20.9	94.8	**<0**.**001**
Skin–epiglottis (cm)	**0**.**923**	0.892–0.947	**>2**.**23**	**90**.**5**	**89.7**	0.802	50.7	98.8	**<0**.**001**
TMD (cm)	0.726	0.680–0.769	**>1**.**05**	66.7	73.9	0.406	23.0	95.0	**<0**.**001**

*ROC curve analysis.

Bold means significant *p* values.

For skin–vocal cord anterior, >1.05 was found to be the cut-off point (AUC = 0.726, *p* < 0.001, 95% CI: 0.680–0.769).

## 4 Discussion

Airway management is a very important issue in terms of the safety of perioperative anesthesia. Complications of difficult airway can have devastating life-threatening consequences in anesthesia management. Therefore, predicting difficult airway is a very valuable step in the planning stage to avoid adverse situations in anesthesia management. Although the importance of ethnicity among airway risk factors is known, inter-ethnic studies with high participation remain limited in the literature. The hypothesis of this study was to determine the clinical and ultrasonographic airway measurements of the Turkish population and to determine and compare the sensitivity, specificity, and cut-off values of these values.

In a clinical meta-analysis study with 35 centers from different countries, the incidence of difficult intubation was found to be 1.5–13% in the general population and 14.3–17.5% in obese patients [11]. In our study, the incidence of difficult intubation was found to be 10% in the general population and 29% in obese patients. Hélène et al. compared 70 obese (BMI ≥ 30) and 61 lean (BMI < 30) patients and found that difficult tracheal intubation was more frequent in obese patients (14.3%) compared to lean patients (3%). They also found that TMD, BMI, large neck circumference, and high Mallampati score were associated with difficult intubation in patients with an IDS greater than 5. They found that neck circumference measurement was a valuable predictive test in both obese and lean patients [12].

In a study conducted by Brodsky et al. at Stanford University with 100 morbidly obese patients, neither absolute obesity nor BMI showed a direct relationship with intubation difficulty. However, they found that potential intubation problems increased as the neck circumference and Mallampati score increased in morbidly obese patients [13]. In a study by Ezri et al. in 50 morbidly obese (BMI > 35) patients, they found that neck circumference measurements were higher in the patient group in whom laryngoscopy was difficult [14].

In our study, we found statistically significant differences between the groups in terms of weight, height, and BMI values. We found that weight (cut-off > 78) and BMI (cut-off ≥ 30.7) values were higher and height values were lower in the patient group with intubation difficulty. Large neck circumference and high Mallampati score were also associated with difficult intubation in our study. There was a positive and statistically significant (*p* < 0.05) correlation between IDS and Mallampati score at the level of *r* = 0.134, with a cut-off value of >38.7 for neck circumference and a sensitivity of 90.5% and specificity of 83.6%.

Baker reported different cut-off values ranging between 6 and 8 cm for TMD measurement [15]. Selvi et al. found that TMD had 2.7% sensitivity and 98.31% specificity at a cut-off value of 6.5 cm and 70.27% sensitivity and 60.87% specificity at a cut-off value of 8.3 cm in their study with 451 Turkish patients [16]. In our study, we found sensitivity and specificity values of 76.2 and 89.4% at a cut-off value of ≤6.8 cm for TMD.

Since there are various cut-off values for TMD and sensitivity and specificity results in studies, new calculations such as height/TMD and neck circumference/TMD ratio, which take into account patient height and body proportions, have started to be used. There are studies showing that these new calculations are more sensitive and specific in predicting difficult intubation. Khan et al. reported that TMD may be insufficient in predicting laryngeal appearance and this value alone is not a sufficient measurement [17]. Therefore, in our study, we wanted to evaluate the relationship between the new evaluation criteria of neck/TMD and neck circumference/TMD ratios with difficult intubation, taking into account that the TMD measurement value is affected by the patient’s height and body proportions. We found the neck circumference/TMD ratio with 92.9% sensitivity and 88.3% specificity at a cut-off value of 5.5, and the neck/TMD ratio with 81% sensitivity and 79.7% specificity at a cut-off value of 24.25 more significant compared to commonly used measurements. We found a positive correlation between the IDS and the height/TMD ratio at the level of *r* = 0.365, and a positive correlation between the neck circumference/TMD ratio at the level of *r* = 0.471. When the two ratios were evaluated within themselves, a positive correlation was observed at the level of *r* = 0.810.

Khan et al. suggested using the ULBT, which evaluates the patient’s ability to close the upper lip mucosa with the lower incisors, in predicting difficult intubation [17]. This method is based on measurements of skeletal hard tissue and soft tissue profiles. Generally, these studies were conducted on Asian and Caucasian populations [17]. Selvi et al. found that the sensitivity and specificity of ULBT in Turkish patients were 18.92 and 99.03%, respectively, and concluded that ULBT may be useful in predicting easy intubation in the Turkish population, but it is not a sufficient test to be used alone in predicting difficult intubation [16]. Kim et al. aimed to evaluate the usefulness of ULBT in predicting difficult intubation in Koreans and found that it showed low false positive rates in Koreans. According to the researchers, these differences are actually due to the lower incidence of high-grade ULBT in Koreans compared to other ethnicities, and this is due to the excess of soft tissue and differences in skeletal structure in Far East Asians [7]. In our study, the rate of patients with ULBT Grade 3 in the IDS Difficult group was found to be similar to other studies with 52%.

In a study conducted in Singapore with 655 patients in which direct laryngoscopic views were evaluated with the Cormack-Lehane scoring system in the Asian population, direct laryngoscopic views of the patients were found as 73.9% Class 1, 21.0% Class 2A, 3.3% Class 2B, 1.6% Class 3, and 0.2% Class 4 [18]. Fernandez-Vaquero et al. found the rates of 58.9% Class 1, 19.1% Class 2A, 17.2% Class 2B, 4.8% Class 3, and 0% Class 4 in a study conducted in Spain in which they evaluated direct laryngoscopic views in 209 patients [19]. In our study, the rates were 58.2% (234 patients) Class 1, 22.4% (90 patients) Class 2A, 10.9% (44 patients) Class 2B, 6.5% (26 patients) Class 3, and 2% (8 patients) Class 4. There was a statistically significant positive correlation between IDS and Cormack Lehane Grade, *r* = 0.726. In our study, when we evaluated the difficult intubation patient group as easy and difficult laryngoscopy (CL Grade 1–2A: easy, CL Grade 2B, 3, 4: difficult), the rate of difficult laryngoscopy in difficult intubated patients was found to be 100%.

When the studies about ultrasonographic evaluations are examined, Ezri et al. found that the ultrasonographically measured skin–vocal cord distance was higher in the patient group in whom laryngoscopy was difficult than in the patient group in whom laryngoscopy was easy. They measured the skin–vocal cord distance in the range of 1.77–2.8 cm in patients with difficult laryngoscopy [14]. In a meta-analysis of 15 studies conducted by Carsetti et al. by searching Medline, Scopus, and Web of Science databases until December 2020, they found that skin–epiglottis, skin–vocal cord, and skin–hyoid bone distances were higher in patients with difficult laryngoscopy than in patients with easy laryngoscopy and that skin–epiglottis distance is the most studied index test in the literature to predict difficult laryngoscopy. They found the sensitivity of skin–epiglottis, skin–vocal cord, and skin–hyoid bone distances as 82, 71, and 75%, and specificity as 79, 71, and 72%, respectively. They determined the distance with the highest specificity and sensitivity as the skin–epiglottis distance [20]. In our measurements, a cut-off value of 0.99 and sensitivity and specificity rates of 66.7 and 70.6%, respectively, were found for the skin–hyoid bone distance, a cut-off value of 2.23 and sensitivity and specificity rates of 89.7 and 90.5%, respectively, for the skin–epiglottis distance, and a cut-off value of 1.05 and sensitivity and specificity rates of 66.7 and 73.9%, respectively, for the skin–vocal cord anterior distance.

Ultrasonographic measurements of the skin–epiglottis distance show differences depending on ethnicity [19]. Adhikari et al. defined a cut-off value of 2.8 cm for skin–epiglottis distance in Caucasian and African-American populations [21], while Wu et al. found a cut-off value of 1.78 cm in the Chinese population [22]. Parameswari et al. found a cut-off value of 1.8 cm in the Indian population [23]. In our study, we found a cut-off value of 2.23 for the skin–epiglottis distance in the Turkish population and sensitivity and specificity rates of 89.7 and 90.5%, respectively.

Numerous studies in different sample groups have shown a relationship between craniofacial skeletal morphology and the degree of sleep apnea [6]. Although Chinese patients are less obese than Caucasians, the prevalence of OSA is similar in the two populations [8]. In a study examining the differences in three-dimensional upper airway anatomy between Asian and European patients with OSA, it was observed that Chinese patients had narrower airways, especially in the retro palatal region, while Icelandic patients had larger tongue, para pharyngeal fat pads, pterygoid, and combined soft tissue volumes [8]. Buxbaum et al. thought that there is an underlying genetic basis for OSA independent of the contribution of BMI [24]. While differences in genetic risk factors may explain disparities in OSA severity, precise anatomical differences have not yet been defined [6]. Asians are thought to be predisposed to OSA because of their craniofacial bone anatomy. Asians with OSA have been shown to have a shorter skull base length than whites [25]. Daraze et al. evaluated the relationship between OSA and airway soft tissue elements and its correlation with gender and anthropometric variables in 117 Lebanese young adults and found that the Lebanese population had shorter soft palate length and their uvula thickness was greater than those reported in Indians compared with Caucasians, Blacks, and Hispanics. They found that the size of the uvula, tongue length, and distances between the epiglottis and the posterior pharyngeal wall were significantly larger in males than in females [26]. In our study, 8% of our patients had a moderate risk of OSA and 8.2% had a high risk of OSA. The number of patients who needed a home continuous positive airway pressure (CPAP) device was eight (2%). When the risk of OSA was evaluated according to gender, no statistically significant difference was found.

## 5 Conclusion

In this study in which clinical and ultrasonographic measurements of the upper airways in the Turkish population were evaluated, we found that the most significant values in terms of both specificity and sensitivity were the skin–epiglottis distance and neck circumference/TMD ratio measured by ultrasonography.

We think that the routine use of body proportions and ultrasonographic methods in preoperative airway assessment, as well as taking into account the ethnicity of the patients, may provide better results in predicting a difficult airway.
